# A Distance Increment Smoothing Method and Its Application on the Detection of NLOS in the Cooperative Positioning

**DOI:** 10.3390/s21238028

**Published:** 2021-12-01

**Authors:** Dongqing Zhao, Dongmin Wang, Minzhi Xiang, Jinfei Li, Chaoyong Yang, Letian Zhang, Linyang Li

**Affiliations:** Institute of Geospatial Information, Information Engineering University, Zhengzhou 450001, China; dongqing.zhao@hotmail.com (D.Z.); xmz276668450@foxmail.com (M.X.); LJF18839128005@163.com (J.L.); ycychaoyong@163.com (C.Y.); zhangletian_1998@163.com (L.Z.); lilinyang810810@163.com (L.L.)

**Keywords:** cooperative positioning, non-line-of-sight error, distance increment, distance smoothing

## Abstract

The wide use of cooperative missions using multiple unmanned platforms has made relative distance information an essential factor for cooperative positioning and formation control. Reducing the range error effectively in real time has become the main technical challenge. We present a new method to deal with ranging errors based on the distance increment (DI). The DI calculated by dead reckoning is used to smooth the DI obtained by the cooperative positioning, and the smoothed DI is then used to detect and estimate the non-line-of-sight (NLOS) error as well as to smooth the observed values containing random noise in the filtering process. Simulation and experimental results show that the relative accuracy of NLOS estimation is 8.17%, with the maximum random error reduced by 40.27%. The algorithm weakens the influence of NLOS and random errors on the measurement distance, thus improving the relative distance precision and enhancing the stability and reliability of cooperative positioning.

## 1. Introduction

Currently, unmanned vehicles, unmanned aerial vehicles, intelligent robots, and other automated platforms have been widely used for military and civilian tasks [1]. The development of swarm intelligence technology inspired by organisms such as bee colonies and ant colonies has promoted research into cooperative task execution using multiple unmanned formations [2]. The advantages of multi–platform cooperation including performance of multiple tasks, high overall efficiency, high system reliability, and strong stability are obvious, and thus, cooperative mission planning among unmanned machines is an area of interest for future development [3,4].

Obtaining accurate positioning information among each platform is an essential prerequisite for performing collaborative tasks. Cooperative localization improves the overall positioning accuracy of the formation using relative navigation information between participants, and it is a vital method for multi–platform formations at present [5,6]. Kurazume et al. [7] first proposed the theory of a cooperative positioning system. In their experiments, three land robots achieved a relative positioning accuracy of 0.4% and an attitude accuracy of 1 degree with the assistance of landmarks and laser ranging. Bahr et al. [8] and Fallon et al. [9] used acoustic ranging to measure the distance between an unmanned surface vehicle (USV) and an autonomous underwater vehicle (AUV) for master–slave cooperative positioning, and they effectively suppressed the AUV underwater navigation error divergence. Chen et al. [1,10] adopted distributed cooperative positioning and used ultra–wideband (UWB) to position unmanned aerial vehicle (UAV) clusters, and the positioning reliability and stability of the formation are significantly improved. Once a multi–platform formation performs a task, it is necessary to avoid obstacles in the surrounding environment, to adjust positions when the task changes, and to repair the blind in the case of individual platform failure. In response to these situations, the formation needs to change and adjust rapidly and safely with accurate formation control. For both multi–platform cooperative positioning and formation control of unmanned platforms, distance information between platforms is a vital piece of the navigational information. Its accuracy is an essential factor in determining the results of multi–platform cooperative positioning and formation control [11].

The most common mechanisms for short–range distance measurement are motion capture, laser, ultrasonic, infrared, ZigBee, and UWB systems. However, motion capture systems are mainly used in indoor environments, laser systems can measure in only one direction, infrared and ultrasonic systems are more easily affected by the external environment, and ZigBee’s ranging accuracy is low. The remaining method, UWB, has strong penetration ability and high ranging accuracy and readily supports networked ranging [12,13]. Therefore, UWB is currently the most popular ranging method in formation environments. However, in a complex external environment, UWB signals are usually blocked by unknown obstacles in the process of transmission, resulting in sizeable random noise and serious non-line-of-sight error (NLOS) error [14].

Existing methods for reducing the distance error are classified as direct or indirect. Direct methods process and restore measured values using the statistical characteristics of the measured values. Direct methods for reducing measurement noise include existing wavelet analysis, Vondrak filters, and Kalman filters. Direct methods for lowering NLOS error include the classical Wylie method, the global migration method, and the two–step estimation method [15]. Indirect methods combine NLOS error elimination with the localization process, with the localization algorithm designed to reduce the influence of NLOS error on the localization results. Indirect methods usually require multiple base stations to obtain location results. This enables NLOS to be detected and estimated according to specific decision criteria. Indirect methods include the weighted least squares algorithm based on optimization theory [16] and a filtering method combining a Gaussian mixture model, extended Kalman filter (EKF), and interacting multiple model (IMM) [17]. However, according to indirect method, we introduce the distance increment (DI) based on the cooperative positioning of an unmanned platform formation to improve the accuracy of distance measurement with the help of the estimated position of each platform in this study.

Our main contributions are as follows. First, we propose a ranging error processing method based on the DI. Second, we reduce the random noise in the distance information and also detect and estimate the NLOS error using our method. Finally, we demonstrate that our method effectively improves the stability and reliability of co–location.

The remaining parts of this article are organized as follows. Section 2 reviews existing research concerning random noise reduction and NLOS. Section 3 first introduces DI and then presents our method for smoothing DI, as well as the distance filtering model and the NLOS estimation method. Section 4 presents our experimental environment and experimental results. Finally, Section 5 summarizes our work directions for future work.

## 2. Related Works

Many scholars have studied mechanisms to reduce the influence of random noise and NLOS error. Methods commonly used to reduce noise include wavelet transforms, Vondrak filters, and Kalman filters. Wavelet analysis is a time–frequency localization analysis method. By decomposing the signal at different frequencies and then eliminating the noise, the spikes and abrupt changes in the signal are preserved. Lin et al. [18] modeled the noise caused by multipath propagation in indoor positioning and used wavelet analysis to remove the noise, effectively weakening the influence of multipath error. Vondrak filters effectively smooth the observed data without knowing the change rule and use a fitting function on the observed data, giving it a wide range of applications in numerical filtering. Zhong et al. [19] used Vondrak bandpass filtering to separate multipath errors and structural vibration information in GPS structural deformation monitoring, thus improving GPS positioning accuracy. Kalman filters take the minimum mean square error as the best estimation criterion to estimate the state parameters and update the state parameters with the prior estimation of the parameters and the new observation data. Using an analysis of the UWB ranging error characteristics, Yin et al. [20] adopted a robust unscented Kalman filter and obtained positioning errors less than 10 cm. Wang et al. [21] applied wavelet analysis, Vondrak filtering, and Kalman filtering to reduce UWB noise in indoor positioning and concluded that Kalman filtering had the best positioning accuracy performance.

In the diagnosis of NLOS, Benedetto et al. [22] and Conti et al. [23] identify NLOS by analyzing received waveforms, but the effect depends on the material and the physical environment. Based on machine learning, Marano et al. [24] used non–parametric regressors to identify NLOS errors, which reduced the influence of environment on the recognition effect, but this method relied on a large number of training data. Xiong et al. [25] used the Cramér–Rao lower bound (CRLB) of cooperative positioning to identify NLOS, which needs more anchors to provide redundant observation information. Landolsi et al. [26] classified different UWB channel models by using the probability density function (PDF) of estimating parameters, such that the recognition accuracy of NLOS can reach more than 90%. Yin et al. [27] designed an iterative algorithm for robust position estimation for the case of unknown PDF, but the computational complexity of this algorithm is greatly improved when the number of distance measurements is small.

The most common method to solve the NLOS error problem is the maximum likelihood method (ML) but obtaining the necessary error and measurement noise distribution is not easy in practice. Guvenc et al. [16] and Picard and Weiss [28] proposed the weighted least square method, which requires measuring only the first and second moments of noise and NLOS errors. Another difficulty is that the machine learning problem is a complex, high–dimensional, non–convex problem, thus obtaining an optimal solution is difficult. Most existing methods apply the relaxation method to the non–convex problem. Some have proposed a semi–definite programming (SDP) relaxation algorithm for cooperative positioning [29,30]. Wang et al. [29] proposed combining the estimated value of the target node location with so–called “equilibrium parameters” to simplify the estimation problem into one solvable with second–order cone programming and applying an SDP algorithm to solve the estimation problem. Biswas [31] proposed graph optimization theory for describing cooperative positioning and deduced the upper and lower bounds of the objective function SDP. Joint estimation involves many optimization variables and requires many accurate measurement data to estimate all parameters accurately. Therefore, optimization–based methods may not perform well in dense NLOS environments due to poor measurement accuracy [32].

Robust filtering methods are another common approach to solving this problem. Based on Kalman filtering, Li et al. [15] proposed the measurement value dropping method, global migration method, and two–step estimation method. These methods effectively eliminated the randomness and positive deviation of NLOS errors in TOA measurements, but the NLOS estimation result depends on the setting of parameters. On this basis, Wang et al. [33] introduced adaptive factors to adjust the parameters, but the algorithm was only suitable for a simple indoor environment and had poor results in a complex NLOS environment. Cui et al. [17] integrated the Gaussian mixture model, extended Kalman filter (EKF), and interacting multiple model (IMM) to overcome the influence of frequent switching between LOS and NLOS environments. The main advantage of this algorithm is that it reduces the influence of severe NLOS in a mixed LOS/NLOS environment. However, the algorithm still needs noise statistics, which are unknown. Chen et al. [34] proposed a robust algorithm using NLOS recognition and classification, dividing NLOS into light and heavy NLOS. The light NLOS was truncated by robust filtering, while the line–of–sight reconstruction estimated the heavy NLOS, but this method relied on an established known platform. Li [2] used the relative velocity between dynamic platforms to detect and compensate for NLOS in the relative navigation to eliminate the dependence on known platforms, effectively improving the detection efficiency. However, this method only estimated NLOS once, resulting in a significant estimation error.

At present, existing work has achieved promising results in reducing the distance error. However, some limitations remain, including the distance noise reduction results affected in the process of dynamic ranging. In addition, accurate estimation of NLOS depends on the layout of known platforms, which limits application scenarios. The relative navigation information between platforms is stable over short intervals, which assists in the noise reduction of distance error and the detection and estimation of NLOS, and the application environment is flexible. Therefore, we propose a range error processing method based on the DI in this study.

## 3. Distance Processing Principle

### 3.1. DI Filtering Model

#### 3.1.1. Cooperative Positioning Model

**Figure 1:**
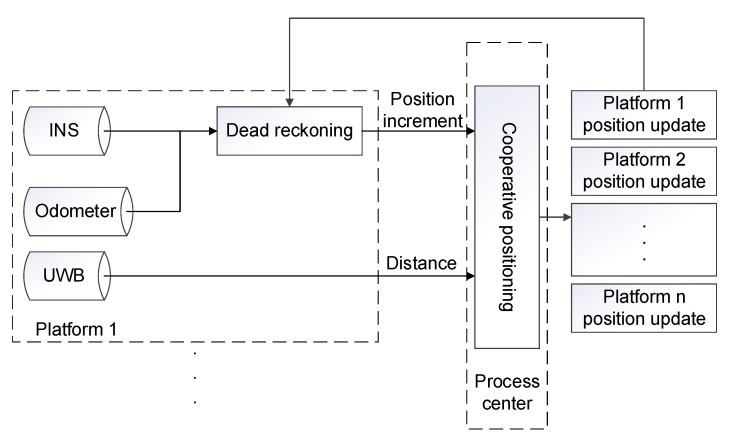
Structure of cooperative positioning algorithm.

In this paper, a hierarchical cooperative positioning algorithm is used. The algorithm structure is shown in Figure 1. Inertial Navigation System/Odometer (INS/OD) integrated navigation is adopted for single platform [35] to carry out dead reckoning, and the obtained position information is transmitted to the processing center according to a certain frequency for interactive fusion. After receiving the position information of each platform, the cooperative positioning processing center uses the distance information to correct the position of each platform. Finally, the processing center transmits the corrected position information to each platform to complete the whole cooperative positioning process. The filtering model of cooperative positioning refers to the model adopted in reference [9].

#### 3.1.2. DI Error Analysis

We first assume that existing platforms 1 and 2 have positions at the time k−1 of $X_{k-1}^{1}\left(x_{k-1}^{1},y_{k-1}^{1},z_{k-1}^{1}\right)$ and $X_{k-1}^{2}\left(x_{k-1}^{2},y_{k-1}^{2},z_{k-1}^{2}\right)$, respectively, with calculated positions of $X_{k}^{1}\left(x_{k}^{1},y_{k}^{1},z_{k}^{1}\right)$ and $X_{k}^{2}\left(x_{k}^{2},y_{k}^{2},z_{k}^{2}\right)$, respectively. The corresponding DI is

(1) $$I_{k}^{12}=\sqrt{{\left(x_{k}^{1}-x_{k-1}^{1}\right)}^{2}+{\left(y_{k}^{1}-y_{k-1}^{1}\right)}^{2}+{\left(z_{k}^{1}-z_{k-1}^{1}\right)}^{2}}-\sqrt{{\left(x_{k}^{2}-x_{k-1}^{2}\right)}^{2}+{\left(y_{k}^{2}-y_{k-1}^{2}\right)}^{2}+{\left(z_{k}^{2}-z_{k-1}^{2}\right)}^{2}}$$

If NLOS are generated during the range measurement at time *k*, the measured distance will change significantly. The measured distance can be expressed as:

(2) $$L_{D_{k}}=L_{D_{k-1}}+I_{k}^{12}+\varepsilon_{NLOS}+e,$$

where $L_{D_{k}}$ and $L_{D_{k-1}}$ are the distance observations; $I_{k}^{12}$ is the distance increment; $\varepsilon_{NLOS}$ is the non-line-of-sight error; e is the measurement error (zero mean Gauss variable). The distance increments from the observations can be expressed as:

(3) $${\bar{I}}_{k}^{12}=I_{k}^{12}+\varepsilon_{NLOS}+e.$$

**Figure 2:**
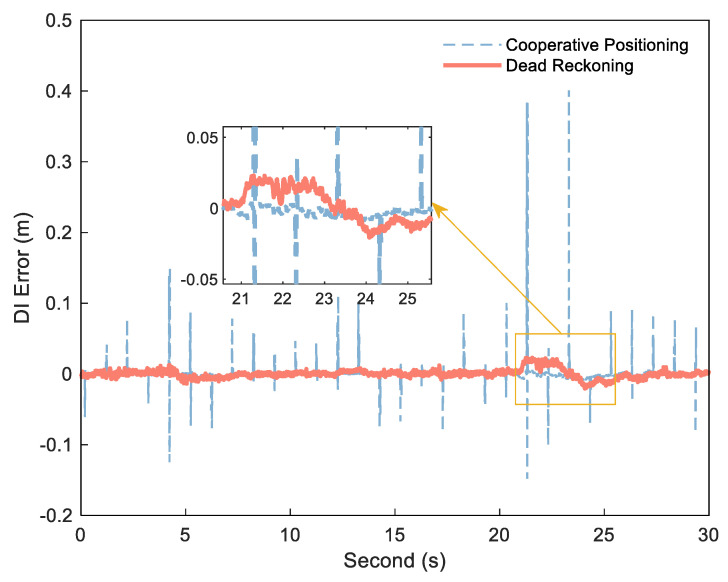
DI error of cooperative positioning (blue dashed) and dead reckoning (red solid).

**Figure 3:**
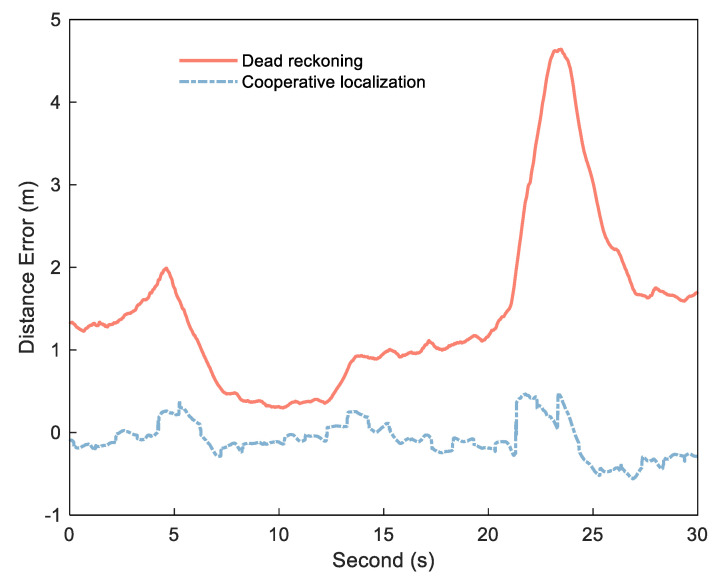
Distance error of cooperative positioning (red solid) and dead reckoning (bule dashed).

If the exact DI is obtained by Formula (1), $\varepsilon_{NLOS}$ can be detected and estimated by ${\bar{I}}_{k}^{12}$ and ${\hat{I}}_{k}^{12}$. The key to obtaining stable, small error range increments is the use of the DI between platform epochs to reduce ranging error. Figure 2 shows the error diagram of the DI calculated by dead reckoning and the DI obtained by cooperative positioning in an experiment, and Figure 3 shows the corresponding distance error. In this experiment, the position of each platform is calculated by Inertial Navigation System/Odometer (INS/OD) integrated navigation system (frequency is 10 Hz). Based on the above system, each platform uses UWB to measure the distance between platforms, and then uses the distance for centralized cooperative positioning (frequency is 1 Hz). The specific experimental environment is described in detail in the follow–up experimental analysis. As can be seen from Figure 2, the DI from dead reckoning is generally stable, but it can be biased in some situations due to the lack of correction for other measurements, as shown in the enlarged portion. In contrast, the error of the DI is small between the two cooperative positionings, but there is a big jump in several epochs after cooperative positioning. The reason of the DI jump in the cooperative positioning is that the estimated value of the platform position will be changed when the cooperative positioning update is performed. As can be seen from Figure 3, these jumps reduce the stability of the DI, but can correct the errors and restrain the speed of error divergence to improve the positioning accuracy in the cooperative positioning.

Therefore, we combine the two kinds of DI and use the DI calculated by dead reckoning to smooth the jump generated by the collaborative update. In this way, we obtain a small error and stable DI.

#### 3.1.3. Filtering Model

According to the characteristics of the two kinds of DI errors, we use the Kalman filter algorithm with additional compensation parameters to smooth the DI. This algorithm with additional compensation parameters adds model parameters to the function model to compensate for model errors and then calculates these additional parameters together with the original state parameters.

By taking the DI from dead reckoning as the predicted value and the DI from cooperative positioning as the observed value, the deviation between the DI values can be estimated. Therefore, the deviation between the dead reckoning DI and the cooperative positioning DI (hereinafter referred to as the DI deviation) is dynamically estimated as an additional unknown parameter vector, which yields the filtering model [36]:

(4) $$\begin{array}{l} \left[\begin{array}{c} I_{k}\\ s_{k} \end{array}\right]=\left[\begin{array}{cc} 1 & 1\\ 0 & 1 \end{array}\right]\left[\begin{array}{c} I_{k-1}\\ s_{k-1} \end{array}\right]+\left[\begin{array}{c} u_{k}\\ 0 \end{array}\right]+\left[\begin{array}{c} w_{I_{k}}\\ w_{s_{k}} \end{array}\right]\\ L_{k}=\left[\begin{array}{cc} 1 & 0 \end{array}\right]\left[\begin{array}{c} I_{k}\\ s_{k} \end{array}\right]+e_{k} \end{array}$$

where $I_{k}$ and $s_{k}$ are the DI and DI deviation, respectively; $L_{k}$ is the observation vector, which represents the cooperative positioning DI; $u_{k}$ is the control vector, which converts the estimated state value at the last time into the dead reckoning DI through the control vector in the filtering process. $w_{I_{k}}$ and $w_{s_{k}}$ are the process noise of DI and DI deviation respectively, assuming that they are zero–mean Gaussian white noise, and the covariance matrix is $Q_{k}$. $e_{k}$ is the observation noise of cooperative positioning DI, assumed to be zero–mean Gaussian white noise with variance $R_{k}$. The variances of $w_{I_{k}}$ and $e_{k}$ can be calculated by the position calculation error of the platform. According to the definition of Formula (1), since the position error at the last moment has no influence on the calculation error, the formula can be abbreviated as

(5) $$I_{k}^{12}=f\left(x_{k}^{1},y_{k}^{1},z_{k}^{1},x_{k}^{2},y_{k}^{2},z_{k}^{2}\right).$$

According to the law of error propagation, the corresponding error expression is as follows:

(6) $${\left(\sigma_{I_{k}^{12}}\right)}^{2}={\left(\frac{\partial f}{\partial x_{k}^{1}}\right)}^{2}{\left(\sigma_{x_{k}^{1}}\right)}^{2}+{\left(\frac{\partial f}{\partial y_{k}^{1}}\right)}^{2}{\left(\sigma_{y_{k}^{1}}\right)}^{2}+{\left(\frac{\partial f}{\partial z_{k}^{1}}\right)}^{2}{\left(\sigma_{z_{k}^{1}}\right)}^{2}+{\left(\frac{\partial f}{\partial x_{k}^{2}}\right)}^{2}{\left(\sigma_{x_{k}^{2}}\right)}^{2}+{\left(\frac{\partial f}{\partial y_{k}^{2}}\right)}^{2}{\left(\sigma_{y_{k}^{2}}\right)}^{2}+{\left(\frac{\partial f}{\partial z_{k}^{2}}\right)}^{2}{\left(\sigma_{z_{k}^{2}}\right)}^{2}$$

After calculating the DI error $\sigma_{I_{k}^{12}}$, we obtain $e_{I_{k}^{12}}=\sigma_{I_{k}^{12}}$. Since the displacement of each platform is equivalent to maintain formation stability in practical application, the difference of position errors in each direction of the platform is ignored. The following formula roughly calculates $\sigma_{I_{k}^{12}}$:

(7) $${\left(\sigma_{I_{k}^{12}}\right)}^{2}={\left(\sigma_{k}^{1}\right)}^{2}+{\left(\sigma_{k}^{2}\right)}^{2},$$

where $\sigma_{k}^{1}$ and $\sigma_{k}^{2}$ are the position drift errors in one direction of platforms 1 and 2 in a given interval and can be calculated based on the accuracy of the sensors in each platform.

In the iterative process of the Kalman filter, the new information vector $V_{k}$ is compared with the preset threshold value. If the new information value is greater than the threshold value, it indicates a jump in the cooperative positioning DI. The dead reckoning DI combined with the DI deviation replaces the state estimate. This method’s threshold value is easy to determine because the jump phase generated by cooperative positioning DI is significant.

### 3.2. Distance Filtering Model

Using the distance and DI between platforms as the state vector and the distance and smoothed the DI measured each time as observations, the filtering model can be expressed as

(8) $$\begin{array}{l} \left(\begin{array}{c} D_{k}\\ I_{k} \end{array}\right)=\left(\begin{array}{cc} 1 & 1\\ 0 & 1 \end{array}\right)\left(\begin{array}{c} D_{k-1}\\ I_{k-1} \end{array}\right)+\left[\begin{array}{c} w_{D_{k}}\\ w_{I_{k}} \end{array}\right]\\ \left(\begin{array}{c} L_{D_{k}}\\ L_{I_{k}} \end{array}\right)=\left(\begin{array}{cc} 1 & 0\\ 0 & 1 \end{array}\right)\left(\begin{array}{c} D_{k}\\ I_{k} \end{array}\right)+\left[\begin{array}{c} e_{D_{k}}\\ e_{I_{k}} \end{array}\right] \end{array}$$

where $D_{k}$ and $I_{k}$ are the distance and DI between platforms, respectively; $L_{D_{k}}$ and $L_{I_{k}}$ are the measured distance and DI after smoothing, respectively; $w_{D_{k}}$ and $w_{I_{k}}$ are the corresponding process noise; $e_{D_{k}}$ and $e_{I_{k}}$ are the related measurement noise.

Since the distance is recursive according to the DI, we set the noise parameter $w_{D_{k}}$ to 0. The process noise is mainly reflected by $w_{I_{k}}$, which is assumed to be zero–mean Gaussian white noise Similarly, assuming that $e_{D_{k}}$ and $e_{I_{k}}$ are zero–mean Gaussian white noise, the variance of $e_{D_{k}}$ can be determined according to the measurement error of the instrument, while the variance of $e_{I_{k}}$ is set to the variance of $I_{k}$ estimated in the previous section.

The relevance of DI with other variables needs to be explained here. It can be seen from Formula (1) that DI is closely related to the velocity of the platform. The greater the velocity of the platform is, the greater the corresponding DI is. In cooperative positioning, the distance is used to correct the platform position, and then the corrected position is used as the observation value to correct the platform’s heading. Therefore, the whole process of cooperative positioning has little influence on the platform’s speed. Here, we can ignore the relevance between DI and distance.

In this filtering model, we use the DI with small and stable error in a short time as the observation quantity to filter the distance measurement value and reduce the influence of measurement noise on the distance value.

### 3.3. Detection and Estimation of NLOS

The method in Section 3.1 enables us to obtain a stable DI with a small error, which can assist in the detection and estimation of the NLOS error. Assuming that the estimated distance value at time k−1 is ${\hat{D}}_{k-1}$, the measured distance value at time k is $L_{D_{k}}$, and the calculated DI is $I_{k}$. The threshold value is then used to determine whether the measured value has NLOS error. We set the threshold as $\gamma_{\max}$ when the following conditions are met:

(9) $$\gamma_{\max}<\left|I_{k}-\left(L_{D_{k}}-{\hat{D}}_{k-1}\right)\right|.$$

Here, the setting of threshold $\gamma_{\max}$ will directly affect the effect of NLOS detection and estimation. If the value of threshold $\gamma_{\max}$ is too small, the normal measurement error will be regarded as NLOS; on the contrary, if the value is too large, the system will ignore some small NLOS. In practical applications, if the ranging accuracy of the rangefinder is known (assuming that the distance variance is $\sigma^{2}$), the probability of observation error less than 2σ is 95.45%, and that less than 3σ is 99.73%. Therefore, if $\gamma_{\max}$ is set to 2σ or 3σ, the corresponding false detection rate is 4.55% or 0.27%. Taking into account the effect of the error of the last time distance estimate, the $\gamma_{\max}$ here is generally set to 3σ. If the ranging accuracy of the rangefinder is unknown, the method of adaptive windowing estimation [37] can be considered to estimate the observation noise of the distance based on the filtering model mentioned in Section 3.2. When the estimated standard deviation is $\sigma^{\prime }$, $\gamma_{\max}$ is still set to $3\sigma^{\prime }$.

We now detail the NLOS error estimation method. Different obstacles have different influences on NLOS errors, but the NLOS errors caused by a single obstacle almost obey normal distribution [38]. In order to reduce the influence of NLOS error, we first estimate the mean of NLOS error and then eliminate it, while the random part can be weakened by filtering. On the basis of the preceding assumptions, the occlusions between the platforms occur at time k, with the measured distance from time k to time k+N containing an NLOS error with mean equal to ε and the time of the NLOS error estimation being set to M. The recursive distance sequence obtained by DI during this period is $D_{k}^{\prime },\;D_{k+1}^{\prime }\cdots D_{k+M-1}^{\prime }$, and the complementary observation distance sequence is $L_{D_{k}},\;L_{D_{k+1}}\cdots L_{D_{k+M-1}}$. We can calculate the NLOS error observation sequence $\varepsilon_{k},\;\varepsilon_{k+1}\cdots \varepsilon_{k+M-1}$ according to the following equation:

(10) $$\varepsilon =L_{D}-D^{\prime }.$$

Since the recursive distance sequence error is small within short intervals, the NLOS error observation sequence can be considered as having equal weight. That is, across duration M of the NLOS error estimation:

(11) $${\hat{\varepsilon }}_{i}=\overset{i}{\underset{j=k}{\Sigma }}\varepsilon_{j}/\left(i-j+1\right)\left(i=k,k+1,\ldots ,k+M-1\right).$$

The final estimate is

(12) $$\hat{\varepsilon }=\overset{k+M-1}{\underset{j=k}{\Sigma }}\varepsilon_{j}/M.$$

**Figure 4:**
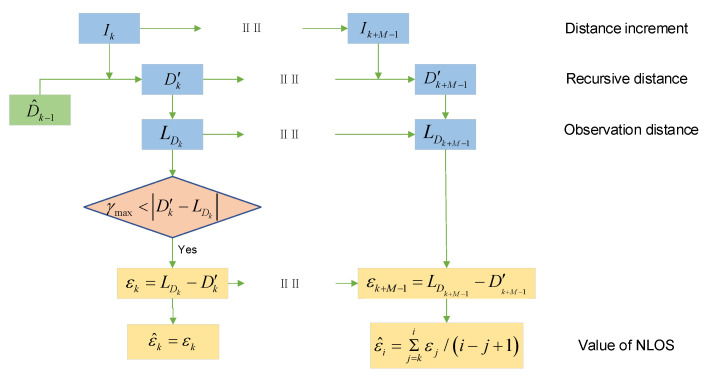
The process of NLOS dynamic estimation.

The process of NLOS error detection and estimation is shown in Figure 4.

### 3.4. Data Processing Flow

Our algorithm has three main parts: the DI smoothing process, the measurement distance smoothing process, and the NLOS error detection and estimation.

1. Using the dead reckoning DI to smooth the jumping part of the cooperative positioning DI to obtain a stable DI with a minor error.
2. Taking the processed DI as the observed quantity to filter and smooth the measured distance.
3. When filtering the observed distance, the NLOS error is detected by the observation residual. If the detection has any NLOS error, the NLOS error is estimated using the range increment and the range observed value.

**Figure 5:**
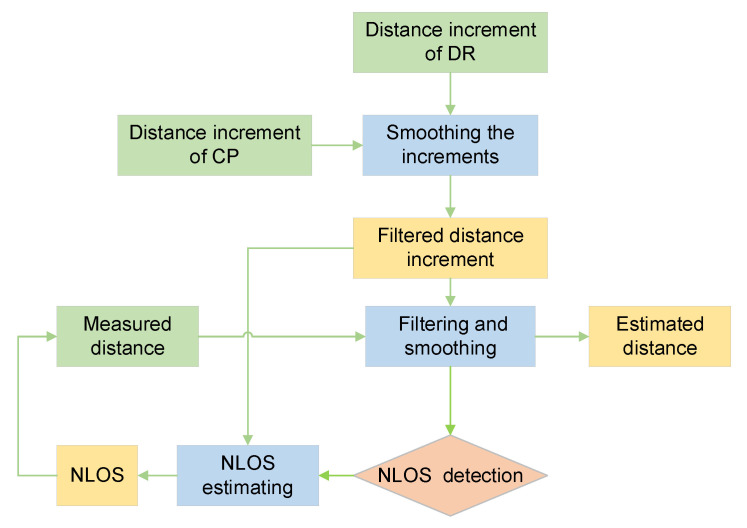
UWB ranging error processing process.

The specific flow chart is shown in Figure 5.

## 4. Experimental Results and Analysis

### 4.1. Experimental Environment Settings

**Figure 6:**
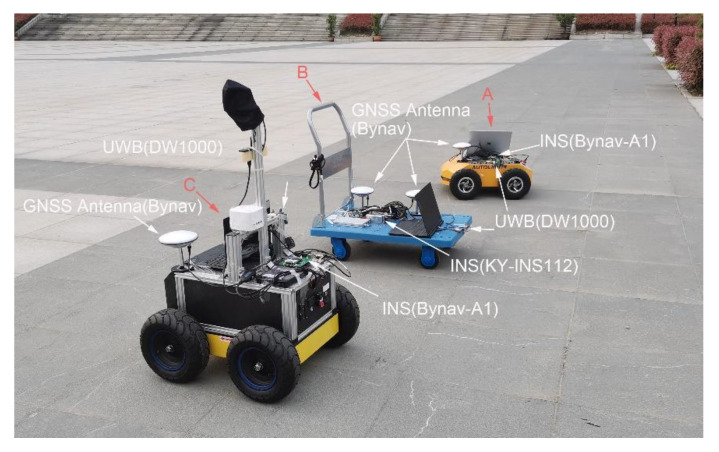
Experimental equipment.

**Figure 7:**
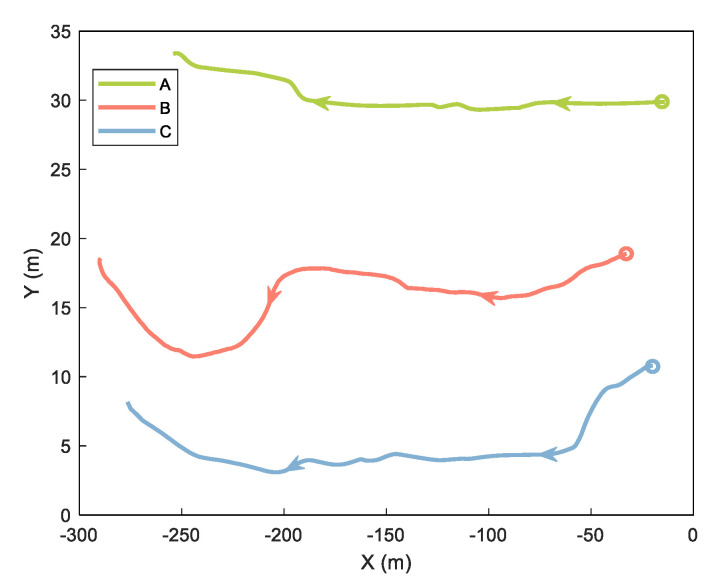
Trajectories of three platforms.

In the cooperative positioning experiment, we used three mobile platforms called A, B, and C. Platforms A and C are robots equipped with odometer, INS, GNSS, and UWB. Platform B is a trolley equipped with INS, GNSS, and UWB. In this experiment, we analyzed the ranging error and verified the proposed method of handling the ranging error. The experimental equipment is shown and labeled in Figure 6. The GNSS base station was placed on the roof of a nearby building about 200 m away. Both cars have built–in odometers. The tracks of the three platforms are shown in Figure 7, with circles representing the starting position and arrows representing the direction of movement.

The full equipment details are as follows.

1. Platform B was equipped with a KY–INS112 module (with a gyro bias less than 0.8°/*h*) in the cooperative positioning system, which is positioned as a pilot using GNSS/INS integrated navigation. Platforms A and C were equipped with Bynav–A1 boards (with a gyro bias less than 2.7°/*h*) positioned as followers using INS/OD integrated navigation. All platforms in the cooperative positioning system made a centralized distance correction once per second.
2. The distance between the platforms was obtained through the DW1000 UWB ranging module (DW1000). The ranging accuracy of the DW1000 is about 10 cm, the two–way ranging accuracy is 15–20 cm, and the ranging frequency is 1 Hz.
3. Keep the trolley in front and the two robots in the back, forming a triangle while driving. The movement speed of the whole formation is maintained at about 0.5 m/s.
4. The robots and trolley were equipped with GNSS receivers able to use PPK results as a reference value for navigation when reprocessing.
5. The pedestrian shielding between the platforms causes NLOS. There were two such shields between Platforms A and B and two shields between Platforms B and C.

### 4.2. DI Smoothing Experiment Results

**Figure 8:**
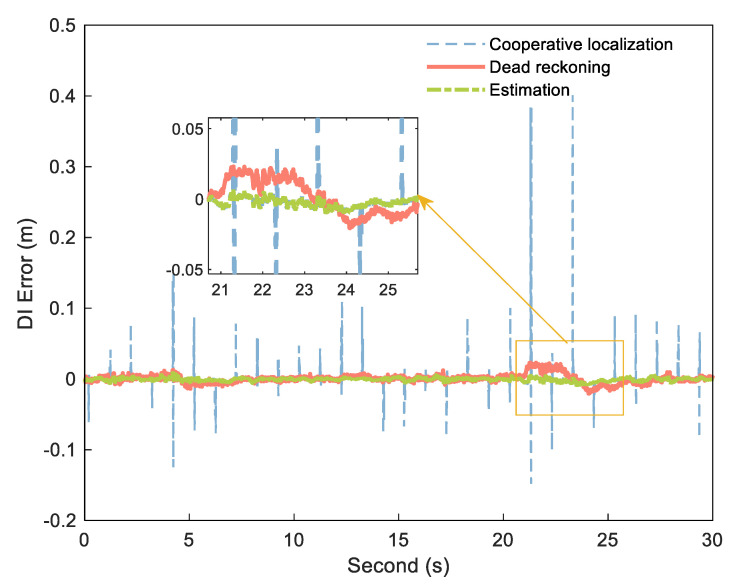
DI error of cooperative positioning (blue dashed), dead reckoning (orange solid), and estimation (green dashdotted).

We selected a measuring distance sequence with sampling time of 30 s between the A and B platforms above and smoothed the cooperative positioning DI using the calculated dead reckoning DI. By comparing the measured data with the reference distance, the system was not affected by NLOS during this period. Figure 8 is the corresponding error curve obtained by subtracting the DI from the reference DI.

As shown in Figure 8, the DI obtained by processing in the above method combines the advantages of the two DIs, with little error and no large jumps.

### 4.3. NLOS Simulation Experiment Analysis

Since it is impossible to obtain accurate distribution of NLOS error in actual environments, our simulation experiment simulated the NLOS error by adding a group of numbers that obey normal distribution to the measured data according to literature [38]. We added a set of numbers distributed as $N(0.45,0.1^{2})$ (NLOS1), $N(0.5,0.1^{2})$ (NLOS2), and $N(0.6,0.1^{2})$ (NLOS3) to the measured distances of 10–20, 50–60, and 90–95 s to simulate the NLOS error. According to our proposed process, we set threshold $\gamma_{\max}$ to 45 cm (three times of ranging error) and set the estimated duration M of NLOS as 3 s. We analyzed the distance error and NLOS estimation time after processing and compared them to the UWB ranging error estimation and compensation methods [2] as a reference method. For ease of description, we call the reference method RN–Based and the DI method DI–Based.

**Figure 9:**
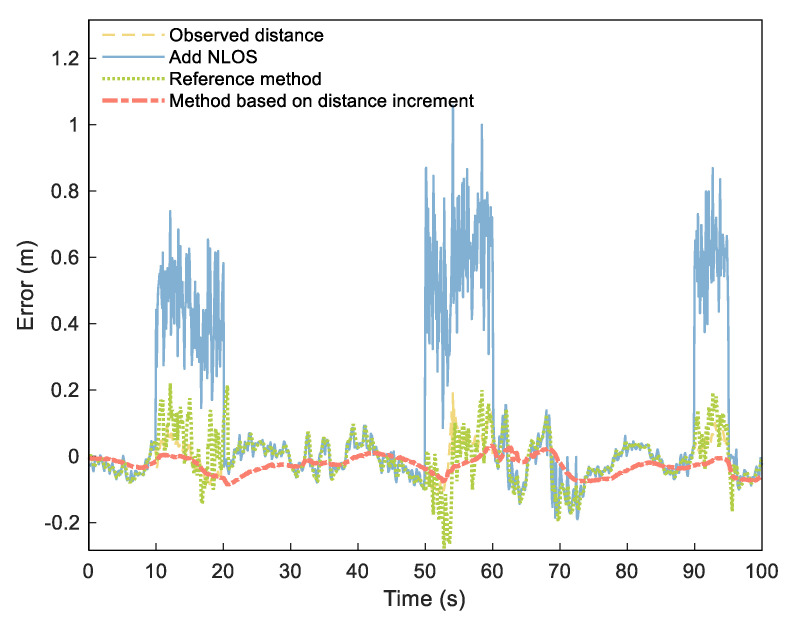
Distance errors of observed distance (yellow dashed), distance adding NLOS (blue solid), RN–Based distance (green densely dotted), and DI–Based distance (orange dashdotted).

Figure 9 shows the range error obtained from the NLOS elimination method. The figure shows that the original observed distance was smoothed to a certain extent, with the NLOS significantly weakened. Table 1 shows the estimation of simulated NLOS values. The three NLOS estimation errors of RN–Based were 0.0517, 0.1031, and 0.0764 m, respectively, and the corresponding error percentages were 11.49%, 20.62%, and 12.73%. However, the estimated errors of the third NLOS calculation of DI–Based were reduced to 0.0368, 0.0067, and 0.0105 m, respectively, with corresponding error percentages of 8.17%, 1.34%, and 1.75%.

**Figure 10:**
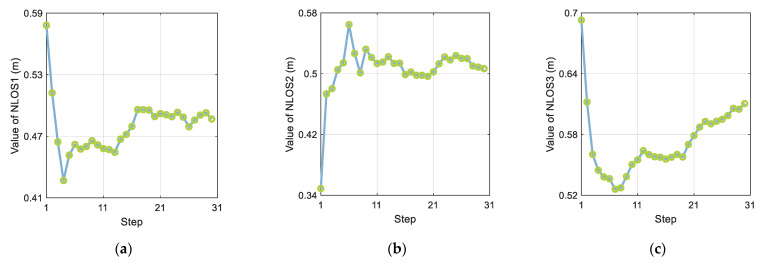
The effect of step size on the three NLOS estimates: (**a**) NLOS1, (**b**) NLOS2, and (**c**) NLOS3.

Figure 10 shows changes in the NLOS estimation process. A total of 30 steps were conducted during the experimental calculation. After 20 steps of the calculation, all three NLOS estimates stabilized. Because the error of the recursive distance sequence increases with time, setting the estimation time interval too large increases the NLOS estimation error and affects the NLOS estimation performance.

### 4.4. Cooperative Positioning Experimental Analysis

**Figure 11:**
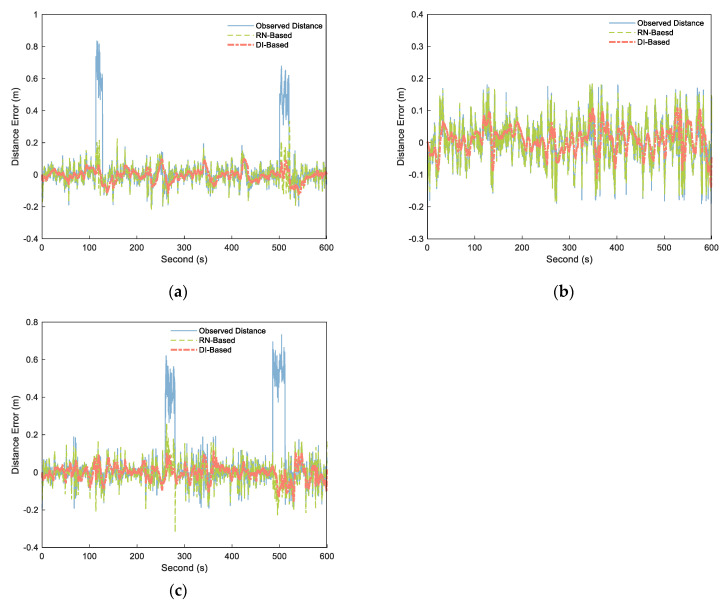
Distance errors of the observed distance (blue solid), RN–Based distance (green dashed), and DI–Based distance (orange dashdotted) over different segments: (**a**) A–B, (**b**) B–C, and (**c**) A–C.

We adopted the preceding distance processing method mentioned above for cooperative positioning and output the distance calculation results of each step. As in the simulation experiment, we set threshold $\gamma_{\max}$ to 45 cm (three times of ranging error) and set the estimated duration M of NLOS as 3 s. By calculate the difference between the output distances and the corresponding distance reference value, as well as the original observation distances and the corresponding distance reference value, the distance errors of the A–B, A–C, and B–C segments were obtained as shown in Figure 11. The four observation range sequences having NLOS errors were successfully detected and modified, and the processed distance weakened the influence of NLOS errors. In the LOS environment, the range fluctuation of the processed distance was minor, and the variation trend of the original observed distance was maintained. In terms of NLOS estimation, the mean values of the two NLOS estimates of A–B segment are 0.5837 and 0.4721 m, and the mean values of the two NLOS estimates of B–C segment are 0.4973 and 0.5468 m. The experiment in literature [38] shows that the mean value of NLOS caused by pedestrians is 0.4–0.6 m, which is consistent with our experimental results.

The data characteristics of each distance sequence were analyzed, including maximum error, mean error, root mean square error (RMSE), signal–to–noise ratio (SNR), and smoothness. Table 2 shows the results. As can be seen from the table, the A–B and B–C segments containing NLOS had a maximum error RN–Based error of 0.3289 and 02653 m and a mean error of −0.0255 and −0.0053 m. The maximum error of the DI–Based was reduced to 0.1024 and 0.1147 m, with the mean error reduced to −0.0059 and −0.0035 m, indicating that the estimation performance of NLOS was better than that of the RN–Based method. For the A–C segment in the LOS environment, the maximum error of RN–Based increased by 2.45%, with no reduction in the mean error. The RMSE fell by 4.88%, the SNR improved by 0.03%, and the smoothness improved by 11.19%. The maximum error, mean error, and RMSE of method 2 fell by 40.27%, 17.07%, and 37.44%, respectively. The SNR and smoothness of method 2 increased by 7.41% and 20.32%, respectively. The DI–Based method had significant improvements in all data characteristics.

**Figure 12:**
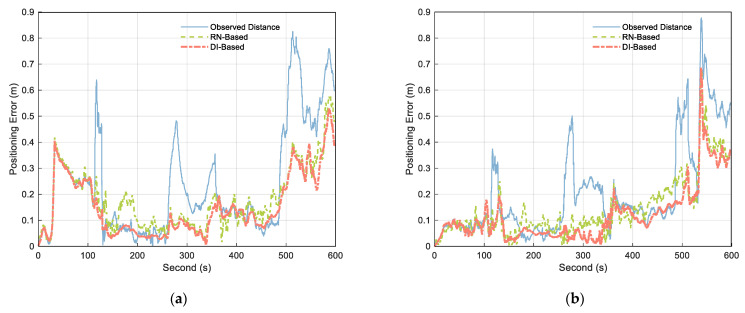
Obtained positioning errors of (**a**) Platform A and (**b**) Platform C using observed distance (blue solid), RN–Based distance (green dashed), and DI–Based distance (orange dashdotted).

Figure 12 compares the positioning error calculated using the original observation distance with that obtained using the treated distance. According to the analysis of the figure, when the measured value contains NLOS, both RN–Based and DI–Based approaches weakened the influence of NLOS, but the DI–Based method was better. The fluctuation range of the positioning error curve shows that the positioning result obtained by DI–Based had fewer fluctuations and the most stable positioning result. This indicates that the method improves the stability and reliability of cooperative positioning.

Table 3 shows the positioning error statistics. According to the mean value of positioning errors in the table, compared with the original observation distance, the errors for Platforms A and C using RN–Based estimation were reduced by 27.05% and 29.39%, respectively. In contrast, those of Platforms A and C were reduced by 35.76% and 40.62%, respectively, under DI–Based. It can be seen that the distance processed by the DI–Based method can be used in cooperative positioning to effectively improve the positioning accuracy, which is consistent with the conclusion in reference [39].

## 5. Conclusions

In this study, we proposed a ranging error processing method using DI to reduce the influence of range error on cooperative positioning and formation control.

By smoothing the DI between platforms, NLOS was detected and estimated by the DI. Smoothing the observed values containing random noise in the filtering process reduced the ranging error.

Simulation and experiment results show the following:

1. Reducing random noise of the range data by introducing the incremental observation of range for filtering significantly improved the processed range for various data features. The maximum value of the random error decreased by 40.27%, and the smoothness increased by 20.32%.
2. In the aspect of NLOS error, the relative accuracy of NLOS estimation was 8.17% by using DI to detect and estimate NLOS continuously.
3. Applying the smoothed distance to the cooperative positioning improved the stability and reliability of the positioning results.

In future research, we intend to focus on the detection of NLOS from the signal level and study relevant fault detection strategies. We will try to provide services for multiple unmanned platforms to position cooperatively in complex environments.

## Figures and Tables

**Table 1:** Estimation of the three simulated NLOS values.

	NLOS1		NLOS2		NLOS3	
Reference value (m)	0.45		0.5		0.6	
Method	RN–Based	DI–Based	RN–Based	DI–Based	RN–Based	DI–Based
Estimated value (m)	0.3983	0.4868	0.6031	0.5067	0.5236	0.6105
Error value (m)	0.0517	0.0368	0.1031	0.0067	0.0764	0.0105
Percentage error (%)	11.49	8.17	20.62	1.34	12.73	1.75

**Table 2:** Data characteristics at different segments.

Segment	Method	Max (m)	Mean (m)	RMSE (m)	SNR (db)	Smoothness
A–B	Observed	0.8348	0.0285	0.1404	42.1575	1.8831
	RN–Based	0.3289	−0.0255	0.0593	49.8267	1.1485
	DI–Based	0.1024	−0.0059	0.0389	53.3832	1.0111
	
A–C	Observed	0.1835	0.0041	0.0641	51.6731	1.2621
	RN–Based	0.1880	0.0041	0.0639	51.6874	1.1209
	DI–Based	0.1096	0.0034	0.0401	55.5046	1.0056
	
B–C	Observed	0.7325	0.0345	0.1438	42.0586	1.6148
	RN–Based	0.2653	−0.0053	0.0608	49.7457	1.1337
	DI–Based	0.1147	−0.0035	0.0404	53.2827	1.0152

**Table 3:** The positioning error statistics of Platforms A and C.

Method	Statistic	Error of A (m)	Error of C (m)
Observed	Max	0.8250	0.8771
	Mean	0.2388	0.2021
RN–Based	Max	0.5874	0.6861
	Mean	0.1742	0.1427
DI–Based	Max	0.529	0.6771
	Mean	0.1534	0.12
